# Supplementary material for: Mid-latitudinal habitable environment for marine eukaryotes during the waning stage of the Marinoan snowball glaciation
Source: Nat Commun. 2023 Apr 4;14:1564. doi: 10.1038/s41467-023-37172-x (PMC10073137; doi:10.1038/s41467-023-37172-x)
Supplement: Supplementary file 1 — Supplementary Information [file 41467_2023_37172_MOESM1_ESM.pdf]

Supplementary Information

Mid-latitudinal habitable environment for marine eukaryotes during the waning stage of the Marinoan snowball glaciation

Huyue Song[1*], Zhihui An[2], Qin Ye[1], Eva E. Stüeken[3], Jing Li[1], Jun Hu[1], Thomas J. Algeo [1,4,5], Li Tian[1], Daoliang Chu[1], Haijun Song[1], Shuhai Xiao[6], Jinnan Tong[1]

[1] State Key Laboratory of Biogeology and Environmental Geology, School of Earth Science, China University of Geosciences, Wuhan 430074, China

[2] Wuhan Center of Geological Survey, Wuhan, 430205, China

[3] School of Earth & Environmental Sciences, University of St. Andrews, St. Andrews KY16 9AL, UK

[4] State Key Laboratory of Geological Processes and Mineral Resources, China University of Geosciences, Wuhan 430074, China

[5] Department of Geosciences, University of Cincinnati, Cincinnati, OH 45221-0013, USA

[6] Department of Geosciences, Virginia Tech, Blacksburg, Virginia 24061, USA

[*]Corresponding author: hysong@cug.edu.cn

Supplementary Note 1. Geological background

The Shennongjia area is located in the northern margin of the Yangtze plate. The first-order structure in this area is a dome-shaped anticline with the middle Proterozoic

Shennongjia Group in the core (Fig. 1b). The Shennongjia Group represents the oldest sedimentary strata exposed in the Shennongjia area. Neoproterozoic strata sit unconformably on the Shennongjia Group and are distributed in a circular belt around the Shennongjia dome. Neoproterozoic strata are unevenly developed in Shennongjia area, and relatively fully developed in the western flank of the Shennongjia dome. These include the Tonian Liantuo Formation; the Cryogenian Gucheng, Datangpo, and Nantuo formations; and the Ediacaran Doushantuo and Dengying formations[1, 2]. On the eastern flank of the Shennongjia dome, strata from the Liantuo Formation to the Datangpo Formation are partially or entirely missing, and the Nantuo Formation directly and unconformably sits on the Shennongjia Group or the Liantuo Formation. In some places, only ~2 m strata of the Nantuo Formation were deposited[3].

The Songluo section (110.5989°E, 31.6854°N) in this study is located in the eastern side of the Shennongjia dome. The Nantuo Formation at Songluo is ~290 m in thickness[4]. It mainly consists of massive diamictite, silty mudstone, and black shale (Fig. 1c). A large number of striated clasts, faceted clasts, and drop stones in Nantuo diamictite strongly suggest its glacial origin[3] (Supplementary Fig. 2). According to the updated stratigraphy of the Nantuo Formation at Songluo[4], carbonaceous compressions[5] and ellipsoidal carbonaceous structures[6] occur abundantly in a black shale bed (here known as the Songluo black shale or SBS, the focus of this study) in the lower Nantuo Formation, although sparse carbonaceous compressions are also found in a dark gray silty mudstone/shale bed in the upper Nantuo Formation (Fig. 1c; Supplementary Fig. 3). Some of these carbonaceous fossils are interpreted as benthic

eukaryotic macroalgae[5]. Thus, their growth and reproduction relied on sunlight for photosynthesis. Although it cannot be completely ruled out that these eukaryotic macroalgae lived in cryoconite pans, which have been proposed as potential refugia for green algae, fungi, protists, and metazoans during the snowball Earth[7, 8], the high organic carbon content of the black shale intervals in the Nantuo Formation indicate that they were unlikely to have been deposited in cryoconite pans where bioproductivity is expected to be low. Instead, these fossiliferous black shales were more likely deposited in a more extensive swath of open water where nutrient supply and bioproductivity were relatively high.

Some researchers argue that the fossiliferous Songluo black shale belong to the Datangpo Formation[9, 10]. However, four lines of evidence strongly indicate that the SBS is part of the Nantuo Formation[3, 4]. (1) The lithology is different between the SBS and the Datangpo Mn-bearing shales in Shennongjia area. In South China, the Datangpo Formation consists of manganese-bearing carbonaceous shale overlain by fine-grained muddy sandstone, muddy siltstone, and mudstone[11]. The basal Datangpo Formation consists of a cap carbonate and Mn-deposits or Mn-carbonate sediments[12]. In the Shennongjia area, the Datapong Formation does outcrop in the western flank of the Shennongjia dome, and it consists of mudstone, siltstone, and Mn-bearing shales, with very high Mn contents (Supplementary Fig. 4b). At the Songluo section in the eastern flank of the Shennongjia dome, the Mn content of the SBS is persistently low. (2) The glacial diamictites above and below the SBS are characteristic of the Nantuo Formation. The Nantuo Formation and the Gucheng Formation are both glacial diamictites, but the

clast composition of these two diamictites are drastically different and easily differentiated in the field (Supplementary Figs. 2, 4). Diamictite of the Gucheng Formation contains sandstone, vein quartz, and chert clasts that are generally small in size (mainly 2 mm – 5 cm in maximum dimension) and relatively sparse in density. In contrast, diamictite of the Nantuo Formation contains abundant granite, gneiss, and carbonate clasts that can reach decimeters in size (mainly 5–50 cm in maximum dimension)[13]. The diamictites underlying and overlying the SBS are similar in clast compositions and both contain clasts characteristic of the Nantuo Formation[3]. (3) Black shale interbeds have been reported from the Nantuo Formation at several sections in South China[14, 15]. At Songluo section, at least two fossiliferous black shale and silty mudstone intervals (i.e., the SBS at ~61 m and a silty mudstone/shale bed at about ~198 m, according to revised stratigraphy of Songluo section)[4, 16], both of which contain carbonaceous macrofossils of the Songluo biota[6], and they are separated by diamictite characteristic of Nantuo glacial deposits. (4) Published geological maps, including those published by refs.[9, 10], show that the Datangpo Formation does not outcrop in study area or in the eastern Shennongjia dome. Instead, the Datangpo Formation is restricted to a small region in western Shennongjia dome, from the Yanduhe town to the Dajiuhu town[1, 17].

Supplementary Table 1. C-N-Fe-Al geochemical data of Songluo section

Sample	Depth		$\delta^{13}C$ org	$\delta^{13}C$ kerogen	$\delta^{15}N$ TN	$\delta^{15}N$ kerogen	TOC	TN	C/N	Fe_{HR}/Fe_T	Fe_{py}/Fe_{HR}	Al	CIA
	m		‰	‰	‰	‰	wt%	wt%	mol/mol			wt%	
SL-1	52.76	diamictite	-28.63		3.77		0.33	0.05	7.78			4.38	60.93
SL-2	54.76	diamictite	-30.75		3.43		1.31	0.54	2.86			4.21	60.74
SL-3	58.76	diamictite	-29.37		3.59		0.38	0.05	8.52			4.17	60.85
SL-4	60.76	diamictite	-29.17		3.42		0.33	0.04	8.92			4.02	59.75
SL-5	61.76	black shale	-28.96	-29.04	5.40	5.49	1.84	0.11	19.43	0.27	0.00	8.53	70.18
SL-6	62.06	black shale	-28.97	-28.92	5.03	5.41	2.28	0.13	21.08	0.46	0.00	8.00	68.75
SL-7	62.36	black shale	-28.99	-28.94	3.96	4.53	4.03	0.14	33.03	0.28	0.00	7.22	64.94
SL-8	62.66	black shale	-29.05	-29.10	3.72	4.33	3.84	0.15	30.55	0.45	0.32	7.32	65.33
SL-9	62.96	black shale	-29.05	-29.11	4.25	4.88	4.43	0.15	34.56	0.58	0.40	7.43	65.82
SL-10	63.26	black shale	-28.99	-29.06	4.98	5.27	4.10	0.14	35.14	0.36	0.48	7.07	65.14
SL-11	63.56	black shale	-29.03	-29.09	4.69	5.14	4.05	0.13	36.86	0.31	0.00	6.88	65.00
SL-12	63.86	black shale	-29.25	-29.26	5.08	5.26	4.10	0.14	33.69	0.29	0.01	7.19	65.88
SL-13	64.16	black shale	-28.97	-29.00	5.27	5.46	3.43	0.14	27.66	0.24	0.00	7.44	66.71
SL-14	64.46	black shale	-29.36	-29.33	4.53	5.04	3.56	0.15	28.52	0.12	0.00	7.46	64.58
SL-15	64.76	black shale	-28.99	-29.03	4.70	5.09	3.91	0.13	34.53	0.18	0.00	7.86	66.98
SL-16	65.76	diamictite	-29.93		3.47		0.31	0.04	8.62			3.88	60.38
SL-17	75.76	diamictite	-28.78		3.21		0.29	0.04	8.94			3.92	60.44
SL-18	77.26	diamictite	-29.62		3.35		0.13	0.05	2.96			3.94	60.31
SL-19	78.76	diamictite	-30.91		3.18		0.28	0.04	7.66			4.02	59.74
SL-20	80.26	diamictite	-28.07		3.84		0.31	0.05	7.39			3.55	60.87

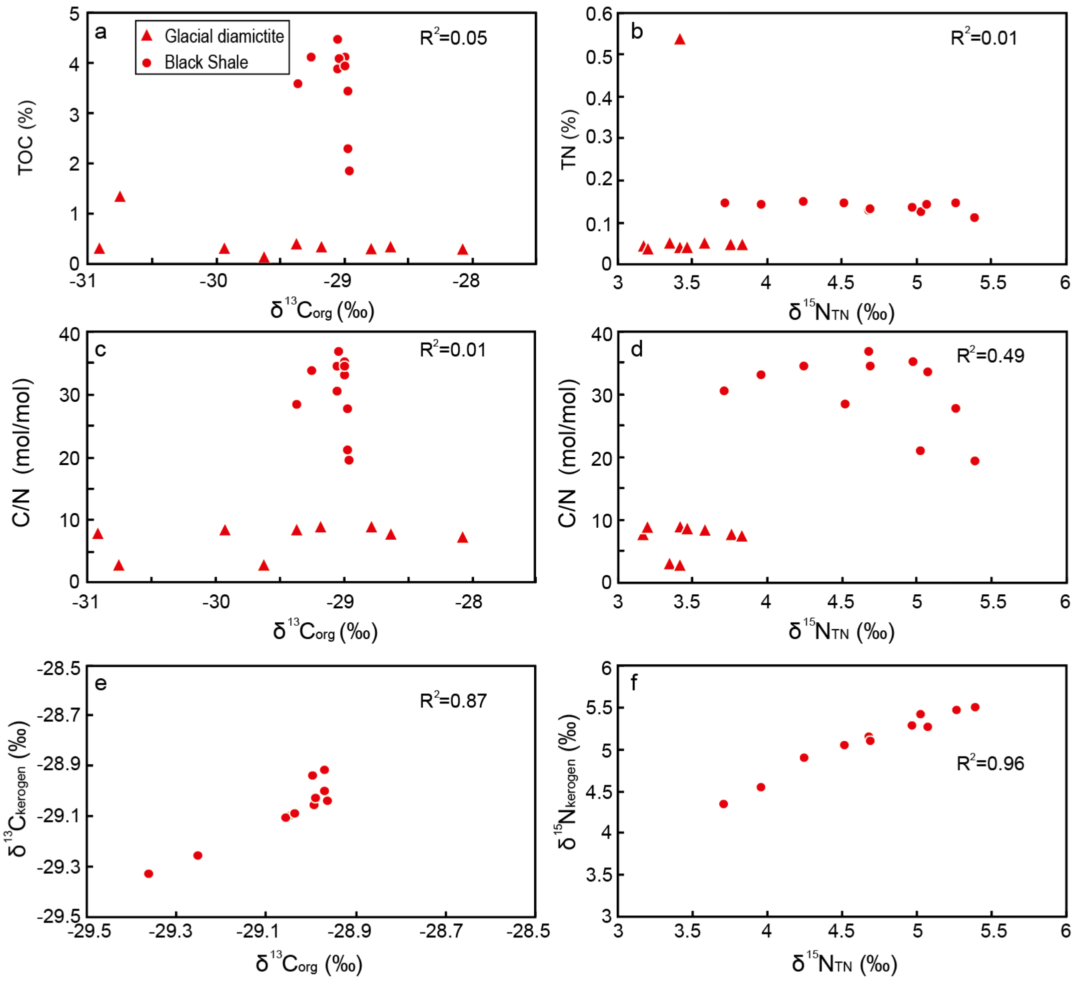

Supplementary Figure 1. Cross-plots of geochemical data. a $\delta^{13}C_{org}$ and TOC. **b** $\delta^{15}N_{TN}$ and TN. **c** $\delta^{13}C_{org}$ and C/N ratios. **d** $\delta^{15}N_{TN}$ and C/N ratios. **e** $\delta^{13}C_{org}$ and $\delta^{13}C_{kerogen}$. **f** $\delta^{15}N_{TN}$ and $\delta^{15}N_{kerogen}$. C: carbon; org: organic; N: nitrogen; TN: total nitrogen; TOC: total organic carbon.

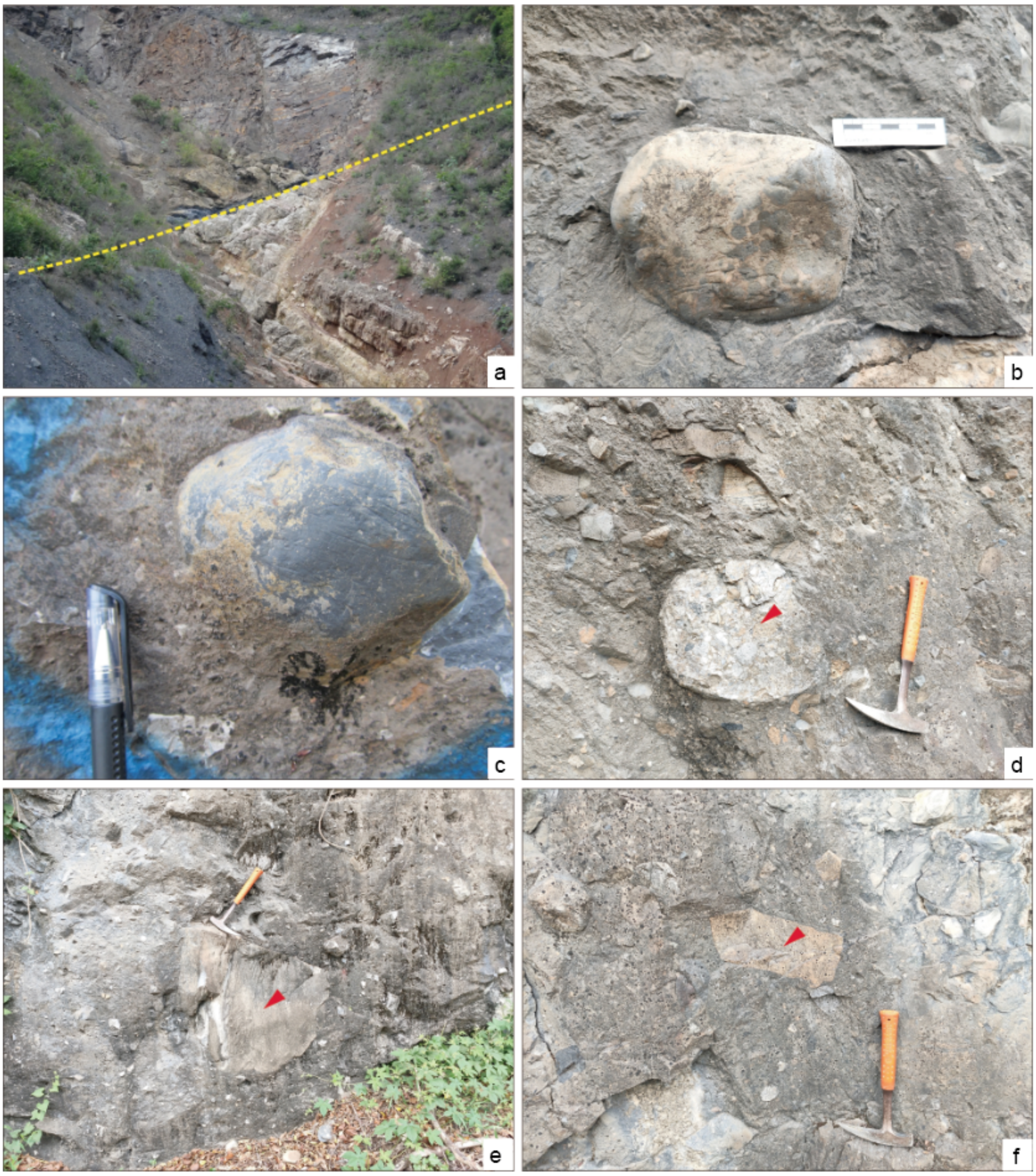

Supplementary Figure 2. Field photos of Nantuo Formation diamictite at Songluo section. **a** Angular unconformity (dashed line) between the Mesoproterozoic Shennongjia Group (lower right) and the Nantuo Formation (upper left). **b–c** Faceted and striated clasts in Nantuo diamictite. **d–f** Clasts of variable sizes in Nantuo diamictite, with red arrows marking gravels. Divisions in Scale bar (b) are centimeters, pen (c) 0.8 cm in diameter, and rock hammer (d–f) is 33 cm in length.

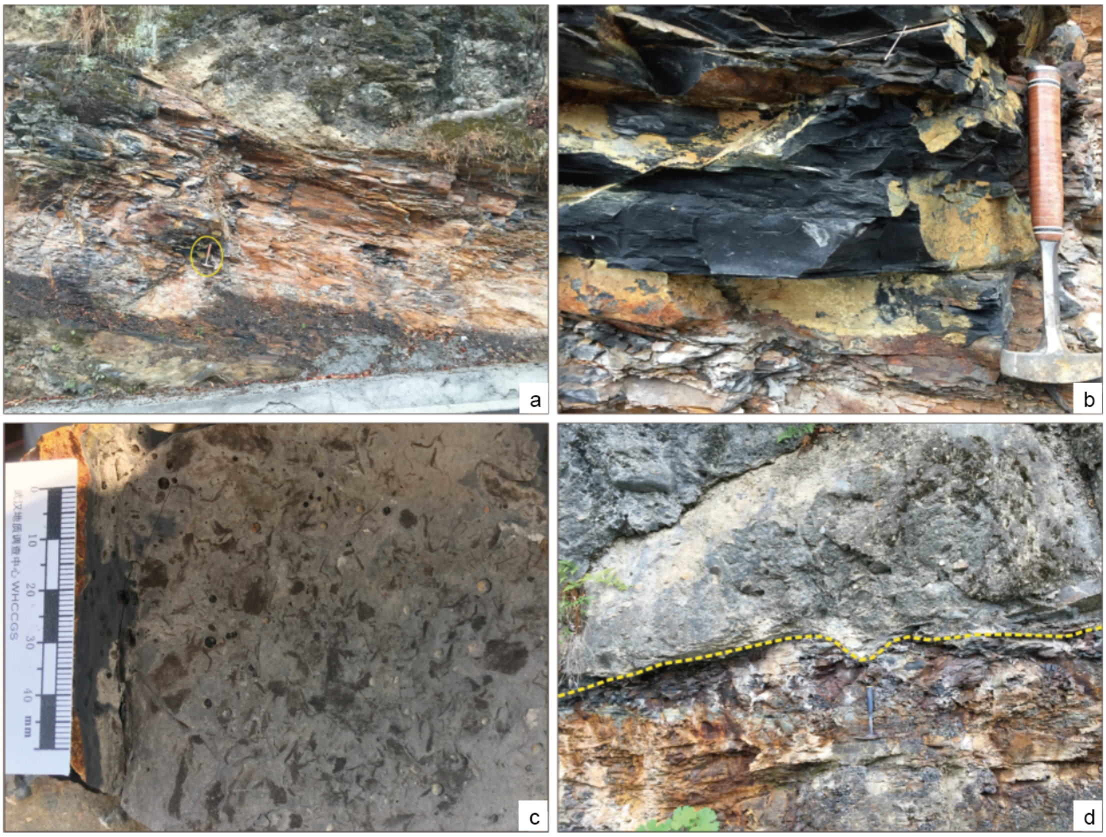

Supplementary Figure 3. Field photos of Songluo black shale (SBS) at Songluo. a SBS sandwiched between Nantuo diamictite with a calcareous matrix. Rock hammer in circle. **b** Close-up view of SBS. **c** Carbonaceous macrofossils preserved in SBS. **d** Contact between SBS and overlying diamictite, as denoted by dashed line. Rock hammer (a–b, d) is 33 cm in length.

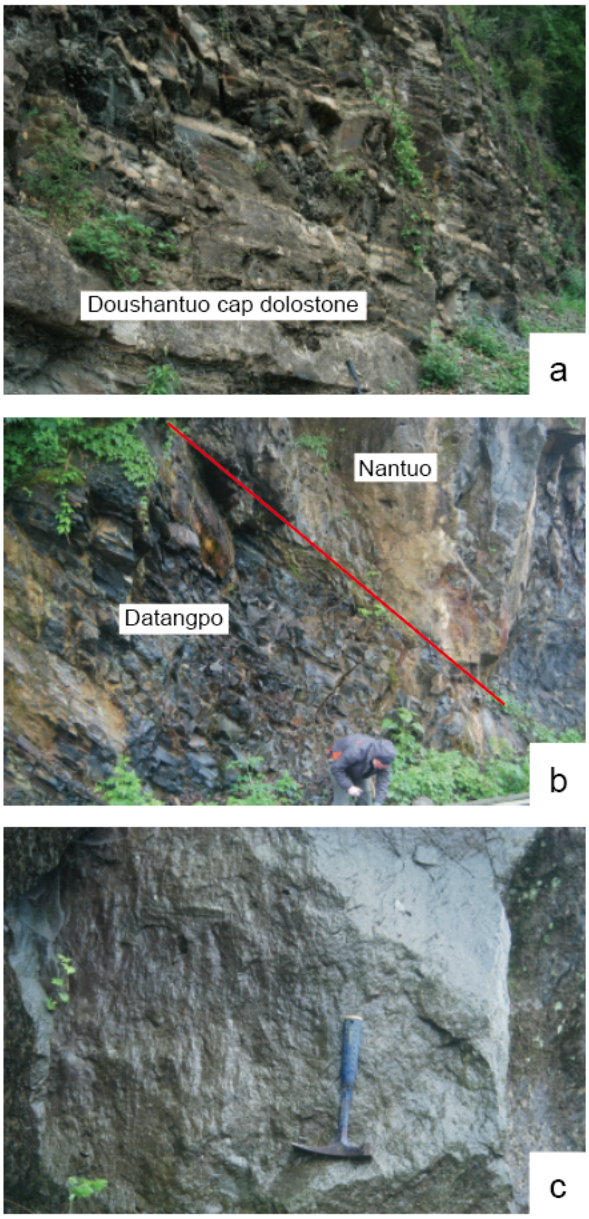

Supplementary Figure 4. Field photographs of Doushantuo, Nantuo, Datangpo and Gucheng Formations in the Shennongjia area. **a** Nantuo-Doushantuo boundary (cap dolostone at rock hammer) at Songluo section. **b** Datangpo-Nantuo boundary in southeastern Shennongjia area. **c** Gucheng diamictite in southwestern Shennongjia area. The man in Fig **4b** is about 1.85 meters tall.

Supplementary References

1. Li, Q. & Leng, J. The Upper Precambrian in the Shennongjia Region. *Science and Technology Publishing House, Tianjin, China*, **565**, 274-278 (1991).

2. Guan, K. et al. Stratigraphic succession of the Nanhua Period in the Shennongjia area in western Hubei and its regional correlation. *Earth Sci. Front.* **23**, 236-245 (2016). (in Chinese with English abstract)

3. Hu, J. et al. Glacial origin of the Cryogenian Nantuo Formation in eastern Shennongjia area (South China): Implications for macroalgal survival. *Precambrian Res.* **351**, 105969 (2020).

4. An, Z., Ye, Q., Hu, J., Tong, J. & Tian L. Stratigraphic position of the Cryogenian Songluo biota in Shennongjia area. *Earth Sci.* doi:10.3799/dqkx.2021.212. (2022). (in Chinese with English abstract)

5. Ye, Q. et al. The survival of benthic macroscopic phototrophs on a Neoproterozoic snowball Earth. *Geology* **43**, 507-510 (2015).

6. Ye, Q. et al. Fossils or sedimentary structures? Carbonaceous spheroids from the shale of the Cryogenian Nantuo Formation in Shengnongjia area, South China. *Precambrian Res.* **345**, 105759 (2020).

7. Hoffman, P. F. Cryoconite pans on Snowball Earth: supraglacial oases for Cryogenian eukaryotes? *Geobiology* 14, 531-542 (2016).

8. Hoffman, P. F. et al. Snowball Earth climate dynamics and Cryogenian geology-geobiology. *Sci. Adv.* **3**, e1600983 (2017).

9. Kuang, H. et al. Benthic macroscopic phototrophs of Cryogenian in Shennongjia that

survived in interglacial period between Sturtian and Marinoan glaciations. *Geol. in China* **44**, 1257-1258 (2017). (in Chinese with English abstract)

10. Chen, X. et al. Revisiting the Nantuo Formation in Shennongjia, South China: A new depositional model and multiple glacial cycles in the Cryogenian. *Precambrian Res.* **356**, 1061132 (2021) .
11. Zhang, Q., Chu, X. & Feng, L. Neoproterozoic glacial records in the Yangtze region, China. *Geological Society London Memoirs* **36**, 357-366 (2011).
12. Yu, W. et al. Newly discovered Sturtian cap carbonate in the Nanhua Basin, South China. *Precambrian Res.* **293**, 112-130 (2017).
13. Nolan, M., Xiao, S., Gill, B., Reid, R. & Schwid, M. Enigmatic provenance of carbonate clasts in Cryogenian glacial diamictite of the Nantuo Formation in South China. *Precambrian Res.* **378**, 106734 (2022).
14. Bao, X. et al. Cyclostratigraphic constraints on the duration of the Datangpo Formation and the onset age of the Nantuo (Marinoan) glaciation inSouth China. *Earth Planet. Sci. Lett.* **483**, 52-63 (2018).
15. Lang, X. et al. Cyclic cold climate during the Nantuo Glaciation: evidence from the Cryogenian Nantuo Formation in the Yangtze Block, South China. *Precambrian Res.* **310**, 243-255 (2018).
16. Ye, Q. et al. Detrital graphite particles in the Cryogenian Nantuo Formation of South China: Implications for sedimentary provenance and tectonic history. *Precambrian Res.* **323**, 6-15 (2019).
17. Lu S. Characteristicis of the Sinian glaciogenic rocks of the Shenongjia region,

Hubei Province, China. *Precambrian Res.* **36**, 127-142 (1987).
